# Objections to the Use of Pessaries

**Published:** 1844-03

**Authors:** 


*Objections to the use of Pessaries.*—The following are the objections to the use of Pessaries, by Dr. Hamilton, long the distinguished Professor of Midwifery, at Edinburg. They are sufficiently intelligible "without note or comment."

*Med. Examiner*, Feb., 1844.

"Firstly, They can only act as palliatives, whatever may be the degree of the disease.

"Secondly, They necessarily keep up a continued irritation in the passage, and of course excite a mucous discharge from the vagina.

"Thirdly, Unless properly adapted, they make injurious pressure on the contents of the pelvis.

"Fourthly, If not frequently taken out and cleansed, they become incrusted with a calcareous matter which proves highly irritating.

"Fifthly, They subject the patient to the charge of a medical attendant for life.

"And lastly, Cases from time to time occur, where, from the laceration of the perineum, &c., no ordinary pessary can be retained."

